# CRAC channel inhibitors in pancreatic pathologies

**DOI:** 10.1113/JP282826

**Published:** 2022-03-16

**Authors:** Oleg V. Gerasimenko, Julia V. Gerasimenko

**Affiliations:** ^1^ Cardiff School of Biosciences, Cardiff University Cardiff CF10 3AX UK

**Keywords:** acute pancreatitis, calcium signaling, CM4620, CM5840, CRAC, pancreatic duct cells

Acute pancreatitis (AP) remains one of the few untreatable inflammatory diseases that requires hospitalization and has a remarkably high mortality rate in severe cases. AP is primarily caused by gallstones and excessive alcohol consumption, but it can also be a side effect of medical treatments, for example with asparaginase (reviewed by Petersen *et al*. 2021). These pathological actions induce excessive cytosolic Ca^2+^ signals, often leading to Ca^2+^ overload, in acinar, stellate, immune and duct cells during the initial stages of AP. Excessive Ca^2+^ release from the internal stores is followed by a subsequent extracellular Ca^2+^ influx through the Orai1 Calcium Release Activated (CRAC) channel, significantly contributing to a sustained Ca^2+^ overload under pathological conditions. Ca^2+^ overload evokes irreversible changes in the pancreas, whereas blockade of such Ca^2+^ elevation has been shown to prevent the development of pathological cellular changes, effectively protecting against experimental AP (recently reviewed by Petersen *et al*. 2021). Since the discovery of the CRAC current by Hoth & Penner (1992) it has been found in many different cell types, including pancreatic acinar cells (Gerasimenko *et al*. 2013). The Orai1 protein has been identified as an essential component of CRAC channels, which are now often referred to as Orai1 CRAC channels.

Under pathological conditions induced by any of the known agents causing AP, the initiating event is extensive Ca^2+^ release from internal stores, which leads to enormous intracellular demand for ATP by the Ca^2+^ extrusion (PMCA) and Ca^2+^ store reuptake (SERCA) mechanisms in a situation with severely reduced ATP production (Petersen *et al*. 2021). The sustained Ca^2+^ overload of the pancreatic acinar cells has the most damaging effect on the pancreas, but other cell types, including duct, stellate and immune cells of the exocrine pancreas, are also involved, creating positive feedback loops magnifying the pathological changes (Petersen *et al*. 2021; Pallagi *et al*. 2022). Excessive Ca^2+^ release under reduced endoplasmic reticulum uptake conditions empties the Ca^2+^ stores, resulting in STIM1 translocation and activation of the Orai1 CRAC channels in the plasma membrane, exacerbating the sustained Ca^2+^ overload. The proof of principle to use inhibition of ORAI1 CRAC channels to alleviate AP was provided by using the CRAC inhibitor GSK7975A by Gerasimenko *et al*. (2013), while CalciMedica (La Jolla, CA, USA) later developed another CRAC inhibitor, CM4620 (recently reviewed by Petersen *et al*. 2021). Both inhibitors have been shown to break the vicious cycle of sustained Ca^2+^ overload in acinar cells as well as in stellate and immune cells, reducing all the hallmarks of AP (Petersen *et al*. 2021).

Pallagi *et al*. (2022) have recently reported the effects of another ORAI1 inhibitor, CM5480 (CalciMedica Inc.) in duct cells, both *in vitro* and *in vivo*, using alcohol‐ or bile‐induced AP (Fig. 1). CM5480 inhibited Ca^2+^ influx in duct cells more slowly as compared to GSK7975A, but it significantly protected the pancreas against damage in a cerulein‐induced experimental mouse model of AP. Protection was less evident in bile‐ and alcohol‐induced AP: CM5480 did not significantly decrease necrosis in bile‐induced AP and did not affect leukocyte infiltration in alcohol‐induced AP. Nevertheless, the paper reports for the first time that inhibition of Orai1 CRAC channels can protect pancreatic ductal cells from sustained Ca^2+^ overload induced by either bile acids or non‐oxidative ethanol metabolites both *in vitro* and *in vivo*. This protection was also sufficient to maintain critical ductal functions, fluid and HCO_3_ secretion, under AP conditions. Since CM5840 only partially inhibited Ca^2+^ influx in duct cells (Pallagi *et al*. 2022), it would be interesting to compare its protective effects with CM4620 (Auxora), an inhibitor that has already reached Phase 2b human trials for AP in the USA.

**Figure 1:**
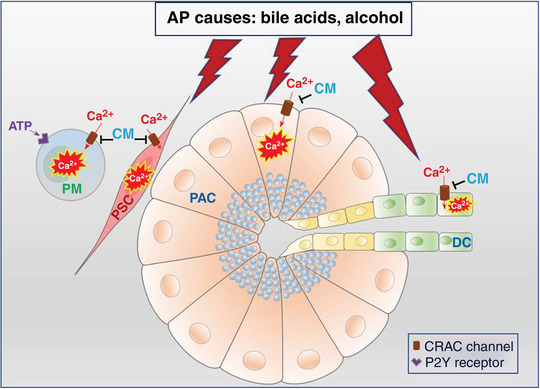
Schematic diagram of the protective effects of inhibition of ORAI1 CRAC channels in bile‐ and alcohol‐induced acute pancreatitis in exocrine pancreas DC, duct cell; PAC, pancreatic acinar cell; PSC, pancreatic stellate cell; PM, pancreatic macrophages.

A graphical representation of the recent findings on the effect of ORAI1 CRAC channel inhibitors in duct cells (DCs), pancreatic acinar cells (PACs), stellate cells (PSCs) and pancreatic macrophages (PM) is shown in Fig. 1. AP inducers such bile acids and alcohol metabolites elicit sustained calcium overload in duct, acinar and stellate cells, leading to necrosis. Proteases, ATP and ADP, released by the damaged cells will affect neighbouring cells, including pancreatic macrophages. Selective inhibitors of CRAC channels, GSK7975A and CM4620, are highly protective against AP in both PACs and PSCs. CM5480 has been shown recently to significantly reduce damage in DCs. Calcium signals in macrophages are usually abolished by the CRAC inhibitors.

Orai1 CRAC channel inhibitors that efficiently reduce Ca^2+^ overload in different cell types of the pancreas are undoubtedly our best hope of developing a cure for the main causes of AP. Another promising development is the use of the energy supplement galactose, which provides cells with additional ATP under AP conditions (reviewed by Petersen *et al*. 2021). However, AP pathologies are not limited to the pancreas. Severe complications can arise in the lungs as a result of the acute inflammatory response. These effects are somewhat similar to those observed in COVID‐19, namely a cytokine storm/inflammatory syndrome. ORAI1 CRAC channel inhibitors can also be used for COVID‐19 pneumonia and COVID‐19 acute respiratory failure (Phase 2b and Phase 2a trials respectively by CalciMedica). In this respect, it is interesting that a recent paper (Gerasimenko *et al*. 2022) reports that the SARS‐COV‐2 S1 spike protein induced Ca^2+^ responses in pancreatic stellate and immune cells *in situ*, which were abolished by the ORAI1 inhibitor CM4620.

There are interesting similarities between cytokine storms in severe cases of AP and COVID‐19 (Petersen *et al*. 2021). New findings provide further arguments in favour of this pharmacological treatment for both AP and severe cases of COVID‐19.

## Additional information

### Competing interests

None.

### Author contributions

Both authors have approved the final version of the manuscript and agree to be accountable for all aspects of the work. All persons designated as authors qualify for authorship, and all those who qualify for authorship are listed.

### Funding

The work was supported by Medical Research Council (UK) (grant MR/J002771/1) and Children with Cancer UK (grants 17/248 and 19/288).

## Supporting information


Peer Review History
