# Supplementary material for: CRAC channel inhibitors in pancreatic pathologies
Source: J Physiol. 2022 Mar 16;600(7):1597–8. doi: 10.1113/JP282826 (PMC9310621; doi:10.1113/JP282826)
Supplement: Supplementary file 1 — Peer Review History [file TJP-600-1597-s001.pdf]

The Journal of Physiology

CRAC channel inhibitors in pancreatic pathologies

Oleg Gerasimenko and Julia Gerasimenko
DOI: 10.1113/JP282826

Corresponding author(s): Oleg Gerasimenko (gerasimenkoov@cardiff.ac.uk)

The following individual(s) involved in review of this submission have agreed to reveal their identity: József Maléth (Referee #1)

Review Timeline:

Submission Date:	02-Feb-2022
Editorial Decision:	08-Feb-2022
Revision Received:	08-Feb-2022
Accepted:	18-Feb-2022

Senior Editor: Kim Barrett

Reviewing Editor: Pawel Ferdek

Transaction Report:

(Note: With the exception of the correction of typographical or spelling errors that could be a source of ambiguity, letters and reports are not edited. Depending on transfer agreements, referee reports obtained elsewhere may or may not be included in this compilation. Referee reports are anonymous unless the Referee chooses to sign their reports.)

1st Editorial Decision 08-Feb-2022

Dear Dr Gerasimenko,

Re: JP-P-2022-282826 "CRAC channel inhibitors in pancreatic pathologies" by Oleg Gerasimenko and Julia Gerasimenko

Thank you for submitting your invited Perspectives article to The Journal of Physiology. It has been assessed by a Reviewing Editor and the author of the focus paper.

Minor alterations have been requested.

The reports are copied at the end of this email. Please address all of the points and incorporate all requested revisions.

NEW POLICY: In order to improve the transparency of its peer review process The Journal of Physiology publishes online as supporting information the peer review history of all articles accepted for publication. Readers will have access to decision letters, including all Editors' comments and referee reports, for each version of the manuscript and any author responses to peer review comments. Referees can decide whether or not they wish to be named on the peer review history document.

I hope you will find the comments helpful and have no difficulty in revising your article within 7 days.

To submit the revised version use the links in Author Tasks Link Not Available.

Please ensure that the article is a Word File with no more than 5 references, including the focus paper.

Thank you for your contribution to the Journal.

Yours sincerely,

Professor Kim E. Barrett
Editor-in-Chief
The Journal of Physiology
https://jp.msubmit.net
http://jp.physoc.org
The Physiological Society
Hodgkin Huxley House
30 Farringdon Lane
London, EC1R 3AW
UK
http://www.physoc.org
http://journals.physoc.org

EDITOR COMMENTS

Reviewing Editor:

Thank you for a very comprehensive Perspectives Article! The Referee suggested only one minor amendment in the text which can be addressed very quickly.

REFEREE COMMENTS:

Referee #1:

This is a very concise summary from real experts of the field, which puts our results into the context of the exocrine pancreatic injury during acute pancreatitis. I only suggest to modify this sentence: "CM5480 was less efficient inhibiting Ca2+ influx in duct cells as compared to GSK7975A". Although the inhibition of the extracellular Ca2+ influx by CM5480 was not as rapid as GSK7975A, but there was no difference in the maximal inhibition. Therefore I think we cannot state the CM5480 was less effective.

Confidential Review 02-Feb-2022

Dear Editor,

Thank you very much for accepting Perspectives article with a minor revision. We have addressed the proposed amendment by changing part of the sentence in question to “CM5480 was inhibiting Ca2+ influx in duct cells slower as compared to GSK7975A”…

Hopefully, this is acceptable.

Best wishes,

Oleg and Julia Gerasimenko.

1st Revision - Editorial Decision 18-Feb-2022

Dear Dr Gerasimenko,

Re: JP-P-2022-282826R1 "CRAC channel inhibitors in pancreatic pathologies" by Oleg Gerasimenko and Julia Gerasimenko

I am pleased to tell you that your invited Perspective article has been accepted for publication in The Journal of Physiology.

NEW POLICY: In order to improve the transparency of its peer review process The Journal of Physiology publishes online as supporting information the peer review history of all articles accepted for publication. Readers will have access to decision letters, including all Editors' comments and referee reports, for each version of the manuscript and any author responses to peer review comments. Referees can decide whether or not they wish to be named on the peer review history document.

The last Word version of the paper submitted will be used by the Production Editors to prepare your proof. When this is ready you will receive an email containing a link to Wiley's Online Proofing System. The proof should be checked and corrected as quickly as possible.

All queries at proof stage should be sent to tjp@wiley.com

Thank you very much for your contribution to The Journal of Physiology.

Yours sincerely,

Professor Kim E. Barrett
Editor-in-Chief
The Journal of Physiology
https://jp.msubmit.net
http://jp.physoc.org
The Physiological Society
Hodgkin Huxley House
30 Farringdon Lane
London, EC1R 3AW
UK
http://www.physoc.org
http://journals.physoc.org

Reviewing Editor Comments:

Thank you for the amendments.

Reviewer Comments:

Referee #1:

The authors addressed the raised minor issue absolutely properly.

1st Confidential Review 08-Feb-2022
